# Activity of terpenes derived from essential oils against *Sarcoptes scabiei* eggs

**DOI:** 10.1186/s13071-021-05094-6

**Published:** 2021-12-09

**Authors:** Meilin Li, Shunlong Liu, Zhijuan Yin, Charlotte Bernigaud, Jacques Guillot, Fang Fang

**Affiliations:** 1 Parasitology Department, College of Animal Science and Technology, Guangxi University, Nanning, China; 2 Dynamic, Ecole Nationale Vétérinaire d’Alfort, Université Paris-Est Créteil (UPEC), 7 Avenue du Général de Gaulle, 94700 Maisons-Alfort, France; 3 Dermatology Department, Faculté de Santé, Hôpital Henri Mondor–Université Paris-Est Créteil, Assistance Publique–Hôpitaux de Paris (AP-HP), Créteil, France; 4 Dermatology Parasitology Mycology Department, Ecole Nationale Vétérinaire , Agroalimentaire et de l’Alimentation (ONIRIS), Nantes, France

**Keywords:** *Sarcoptes scabiei*, Scabies, Essential oil, Terpenes, Ovicidal activity

## Abstract

**Background:**

The limited ovicidal activity of currently available acaricides is a significant obstacle to efficacious scabies treatment. Several essential oils or their respective components have proved to be active against the eggs of arthropods, mainly lice and ticks. Information on the activity of these oils and/or components against the eggs of mites remains very limited. The aim of this study was to assess the activity of six terpenes (carvacrol, eugenol, geraniol, citral, terpinen-4-ol and linalool) commonly found in essential oils against the eggs of *Sarcoptes scabiei*.

**Methods:**

*Sarcoptes* eggs were exposed to paraffin oil containing 1, 2.5, or 5% of each terpene tested. After a 12-h exposure period, the eggs were washed and placed in paraffin oil for hatching. Embryonic development following treatment was assessed every day to determine the stage of developmental arrest.

**Results:**

The median effective concentration to obtain 50% egg mortality (EC_50_) was 0.5, 0.9, 2.0, 4.8, 5.1 and 9.8% for carvacrol, eugenol, geraniol, citral, terpinen-4-ol and linalool, respectively. The microscopic images of eggs after each treatment indicated that these six terpenes may act by penetrating through the aeropyles on the egg surface.

**Conclusions:**

In conclusion, carvacrol, eugenol and geraniol possess significant ovicidal activities, which should be considered as promising ovicidal agents for the treatment of scabies.

**Graphical Abstract:**

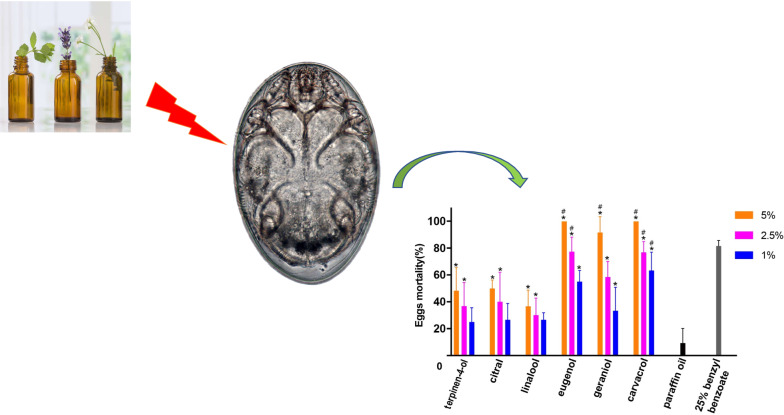

## Background

*Sarcoptes scabiei* is a parasitic mite responsible for the skin disease called scabies. With an estimated 200 million people infected worldwide, scabies is one of the most prevalent infectious skin diseases [1]. It remains a major public health issue in many resource-poor areas. In addition to being a highly contagious skin infestation, scabies can induce secondary bacterial infections and potentially life-threatening sequelae, including sepsis, post-streptococcal glomerulonephritis, rheumatic fever and heart disease [1].

Relatively few treatments are currently available for the management of human scabies; these include a number of topical agents, such as 5% permethrin (pyrethroid), 10–25% benzyl benzoate, crotamiton (aniline) or 0.5% malathion (organophosphorate), and one oral treatment (ivermectin [macrocyclic lactone]) [2]. The limited ovicidal activity of these currently available acaricides has been and remains a significant obstacle for efficient scabies control. All treatments require a second administration of the agent after a 7- to 14-day interval to kill newly hatched mites, but poor patient compliance with repeated treatments can lead to treatment failure [3, 4]. Therefore, there is an urgent demand for novel drugs which can target the eggs of *S. scabiei*.

*Sarcoptes* eggs are oval in shape and exhibit numerous aeropyles on their external surface. The egg shell consists of two layers: an inner translucent layer and an outer layer of finger-like projections with rounded tips [5]. Bernigaud et al. [4] described two phenotypes of egg stage: (i) an early embryonic neurologically immature and immobile accumulation of cells with an absence of a differentiated nervous system; and (ii) a mature embryo, still inside the egg, with the same vital systems found in newly emerged larva, including nervous, respiratory and circulatory systems. The early embryonic stage of *Sarcoptes* eggs as drug targets appears to be unexplored so far.

Essential oils and their constituents represent an appealing alternative strategy against *S. scabiei*. Essential oils of tea tree (*Melaleuca alternifolia*) [6], lemongrass (*Cymbopogon citratus*) [7], lavender (*Lavandula angustifolia* Mill.) [8], clove (*Eugenia caryophyllata*) [8, 9] and palmarosa (*Cymbopogon martinii*) [8] have been shown to possess a strong activity against the motile stages (larvae, nymphs and adults) of *S. scabiei*, with complementary studies demonstrating that their major components, terpinen-4-ol and eugenol, were also effective against *Sarcoptes* mites [6, 9]*.* The terpene citral, linalool, geraniol and carvacrol were shown to have a significant acaricidal effect on *Psoroptes* mites and eggs [10, 11], with supporting evidence that these terpenes may also be active against the eggs of mites [10]. The objective of the present study was to assess the effect of terpinen-4-ol, citral, linalool, eugenol, geraniol and carvacrol on the eggs of *S. scabiei*.

## Methods

The *Sarcoptes* eggs used in this analysis were collected from the crusts of naturally infested New Zealand White rabbits kept on a rabbit farm in Nanning, Guangxi Province, China. Prior to initiating the study, the farm owners were contacted and permission subsequently obtained to use the infected rabbits for the purpose of the study. The crusts were scraped from the skin of the infected rabbits with a scalpel, placed in Petri dishes and then transported to the laboratory within a few hours. *Sarcoptes* eggs were isolated individually using a needle for testing under a stereomicroscope (SMZ745; Nikon Corp., Tokyo, Japan; 2× magnification).

Six terpenes (terpinen-4-ol, citral, linalool, eugenol, geraniol and carvacrol) were purchased from Shanghai Macklin Biochemical Company (Shanghai, China). All compounds were of the highest purity available (purity 95–99%). To test the ovicidal activity of these compounds, *Sarcoptes* eggs at the early embryonic stage were placed on a microscope slide and exposed to paraffin oil containing 1, 2.5 or 5% of each terpene. After a 12-h exposure period, the eggs were washed and placed in paraffin oil for subsequent hatching. Eggs exposed to 25% benzyl benzoate (Aladdin, Shanghai, China) were the positive control group, and eggs exposed only to paraffin oil (Aladdin) were the negative control group. The eggs were incubated in a humidity chamber (≥ 70% relative humidity) at 35 °C for 5 days. Six replicates (each of 10 eggs) were performed for each terpene and each concentration.

To detect and describe changes in the development of the embryos, eggs at the early embryonic stage were exposed to 1% of each terpene and placed on a microscope slide. The development of these eggs in paraffin oil and in 25% benzyl benzoate was also assessed as controls, as was the effect of 25% benzyl benzoate separately. A normal development was obtained with eggs in paraffin oil. At least five eggs were used for each solution. The slides were incubated in a humidity chamber (≥ 70% relative humidity) at 35 °C for 72 h, following which the eggs were examined under a microscope (Eclipse 80i; Nikon Corp.; 40× magnification) and pictures taken every 12 h. During this process, the development of embryos in the eggs was observed. Eggs were considered dead if they failed to hatch after 72 h.

Results were analyzed using SPSS software version 20.0 (SPSS IBM Corp., Armonk, NY, USA). The median effective concentration to obtain 50% egg mortality (EC_50_) was calculated by probit regression analysis. Data were analyzed with one-way analysis of variance followed by least significant difference. Values of *P* < 0.05 were considered significant.

## Results

**Fig. 1:**
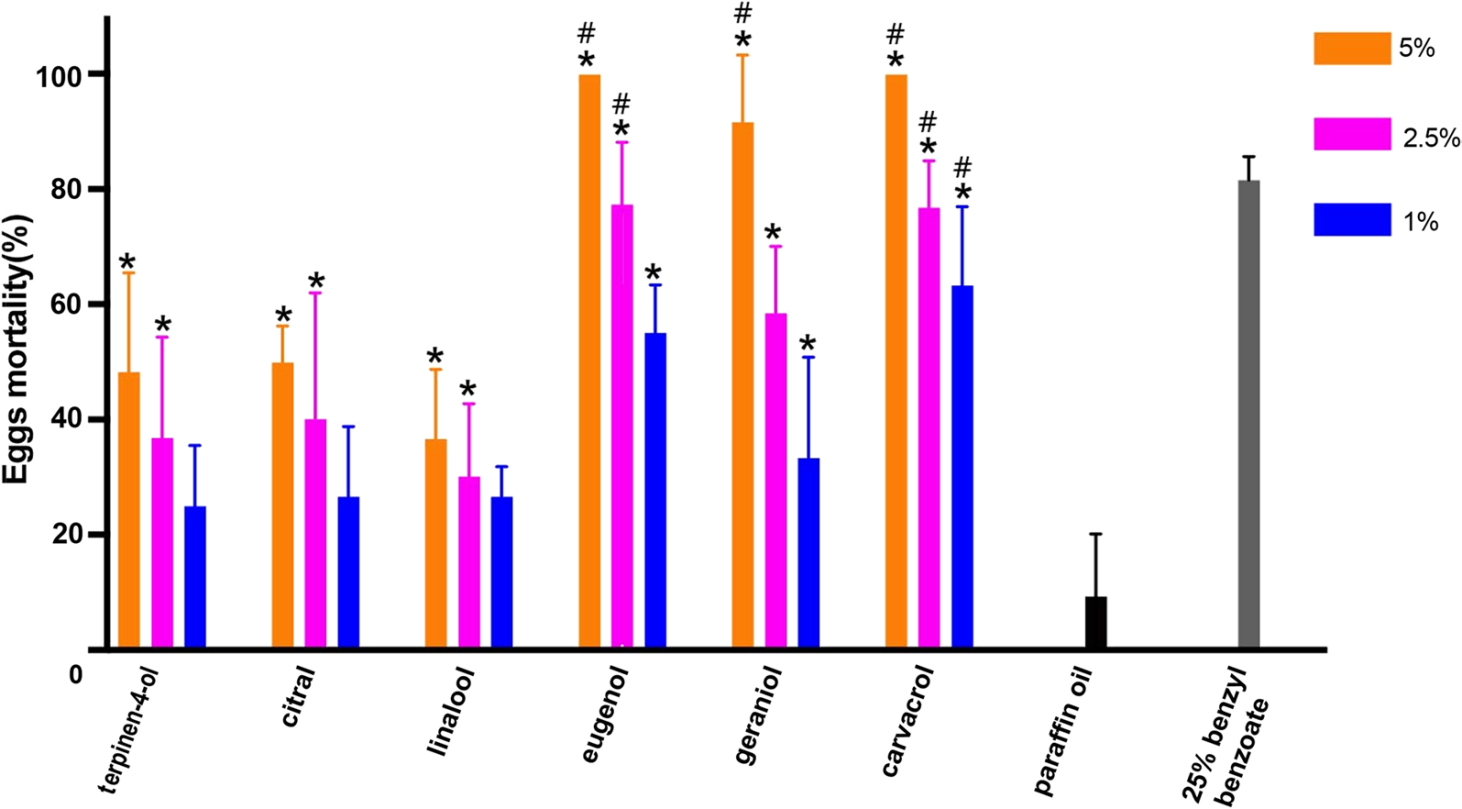
Mortality rate of *Sarcoptes* eggs exposed to six terpenes at a concentration of 1, 2.5 and 5%, respectively. Asterisk above each bar indicates a significant difference (*P* < 0.01) in egg mortality between the indicated concentration of terpene and paraffin oil (negative control). Hash sign above bar indicates a significant difference (*P* < 0.01) in egg mortality between the indicated concentration of terpene and 25% benzyl benzoate (positive control)

At the test concentration of 5%, the ovicidal effect of carvacrol, eugenol, geraniol, citral, terpiene-4-ol and linalool was 100, 100, 91.7, 50.0, 48.3 and 36.7%, respectively (Fig. 1). A mortality rate of 81.7% and 7.8% was observed in the positive control group (eggs exposed to 25% benzyl benzoate) and the negative control group (non-treated eggs), respectively. Compared to the negative control, exposure to the six terpenes at the concentrations of 5% and 2.5%, as well as exposure to carvacrol and eugenol at the concentration of 1%, led to significant differences in *Sarcoptes* egg hatching rate (*P* < 0.01). There was no significant difference (*P* > 0.5) in terms of hatching rate between eggs in the positive control group and those exposed to all concentrations of carvacrol, eugenol at concentrations of 2.5% and 5% and geraniol at the 5% concentration. The EC_50_ value was 0.5, 0.9, 2.0, 4.8, 5.1 and 9.8% for carvacrol, eugenol, geraniol, citral, terpinen-4-ol and linalool, respectively (Table 1). Embryos in eggs exposed to 1% terpinen-4-ol (Table 2, images 8–11) those in eggs exposed to linalool (Table 2, images 16–19) developed normally and larvae hatched within 72 h. Embryos in eggs exposed to 1% citral (Table 2, images 12–15) developed to a certain stage but ultimately stopped developing; embryos in eggs exposed to 1% carvacrol (Table 2, images 26–28), 1% eugenol (Table 2, images 20–22), 1% geraniol (Table 2, images 23–25) and 25% benzyl benzoate (Table 2, images 5–7) did not develop by 24 h, and the eggs remained at the early embryonic stage (Table 2). Eggs shells in all treatment groups appeared to be intact.

**Table 1:** Probit regression analysis of the activity of the six terpenes against *Sarcoptes scabiei* eggs

Compounds	EC_50_^a^ (95% confidence limits)	Pearson Chi-square
Terpinen-4-ol	5.1% (3.8–12.2)	15.8
Citral	4.8% (3.5–11.5)	15.2
Linalool	9.8%^b^	7.6
Eugenol	0.9% (0.1–1.4)	9.2
Geraniol	2.0% (1.4–2.4)	18.5
Carvacrol	0.5% (0.7–1.1)	9.2

^a^Activity of tested compound was determined based on the median effective concentration to obtain 50% egg mortality (EC_50_)

^b^95% confidence limits were not available

**Table 2:** Microscopic aspect of *Sarcoptes* eggs at different time points after exposure to terpinen-4-ol, citral, linalool, eugenol, geraniol and carvacrol over time

Treatment groups	0 h	24 h	48 h	72 h
Paraffin oil (negative control)				
25% Benzyl benzoate (positive control)				
Terpinen-4-ol				
Citral				
Linalool				
Eugenol				
Geraniol				
Carvacrol				

## Discussion

The results of the present investigation demonstrated for the first time the strong ovicidal activity of several terpenes extracted from essential oils against the eggs of *Sarcoptes scabiei*, of which carvacrol, eugenol and geraniol showed the highest activity. The ovicidal activity of carvacrol has been reported in ticks, with a hatching rate of 8.3% at 1% concentration [12]. The ovicidal activity of eugenol, geraniol, citral, terpinen-4-ol and linalool has been reported against *Psoroptes* eggs [10] and is consistent with the activity against *Sarcoptes* eggs demonstrated in the present study. The same terpenes have also been shown to have an effect on the eggs of lice [13, 14] and mosquitoes [15].

Many essential oils and/or their components have been shown to be neurotoxic, acting on different targets in the nervous system of arthropods. It has been proven that terpinen-4-ol, linalool, carvacrol and geraniol inhibit the activity of acetylcholinesterase in different insects (including head lice) and also ticks [16–18]; linalool and carvacrol interfere with γ-aminobutyric acid receptors in insects [17, 19]; eugenol, geraniol, carvacrol and citral block the octopamine receptor binding sites in insects [20, 21]; and eugenol interferes with cell membranes and organelles in epidermal and gut epithelia of *Sarcoptes* mites [9]. Information on the mechanisms of action of essential oils and/or their components on the eggs of arthropods is much more limited. In the present study, serial microscopic examination revealed that unhatched eggs, especially embryos in eggs exposed to carvacrol, eugenol and geraniol, ceased to develop within 0  and approximately 24 h and that the eggs remained in the early embryonic stage (Table 2). The ovicidal action of these terpenes is due to their ability to penetrate into the eggs, possibly through aeropyles on the surface of *Sarcoptes* egg shells [22]. Conversely, the lack of ovicidal activity of a chemical may be due to its poor penetration into the eggs. Active terpenes can kill eggs in the early embryonic stage. In our study, embryos in eggs exposed to those terpenes showing less ovicidal activity (linalool, citral, terpinen-4-ol) remained half-developed or were premature, but the eggs ultimately hatched, suggesting that these terpenes may not be neurotoxic to the mites.

In clinical practice, topical agents for the treatment of scabies should be applied onto the skin surface of patients and left for 8–12 h [23]. In the present study, we exposed the eggs to each solution for only 12 h, whereas the classical technique with a filter paper results in an exposure duration of up to several days. The classical technique consists of placing a filter paper at the bottom of a Petri dish and impregnating it with a test solution; eggs are then placed on the filter paper and exposed to the test solution during the whole process [10]. With a 12 h-exposure period, 19.3% of eggs were able to hatch when exposed to 25% benzyl benzoate, whereas only 8.3% of eggs finally hatched with the filter paper method [7]. In the present study, only carvacrol, eugenol or geraniol led to > 90% mortality rate of *Sarcoptes* eggs after 12 h exposure to the 5% solution. In preliminary tests with the filter paper method, we observed that all of the six terpenes (when used at the 5% concentration) were able to kill > 90% eggs (unpublished data). These findings suggest that carvacrol, eugenol and geraniol can act on eggs with a short contact time.

*Sarcoptes* mites are difficult to sample in large numbers from human patients, and no in vitro culture system has yet been established [3]. Mites from different hosts may exhibit minor differences in terms of morphology and host preference, but they share the same biology [24]. From previous studies, it can be postulated that there is no difference in the survival rate of *Sarcoptes* eggs from animal hosts and humans under the conditions tested [3].

The main limitation for the use of essential oils or related compounds (i.e. terpenes) is the risk of a skin reaction [25]. However, by limiting the dose and concentration, we can prevent essential oils from causing significant health risks. Tisserand and Young [25] recommend that the maximum dermal use level for carvacrol, eugenol and geraniol is 1, 0.5 and 5.3%, respectively. The EC_50_ was 0.5, 0.9 and 2.0% for carvacrol, eugenol and geraniol, respectively. Therefore, the use of topical geraniol and carvacrol should be safe and be expected to have a satisfactory ovicidal effect against *S. scabiei.*

## Conclusions

In conclusion, carvacrol, eugenol and geraniol possess significant ovicidal activities. The results of the present study provide a scientific basis for the application of essential oil and/or their constituents as a therapeutic agent for scabies. Further studies should consider testing combinations of terpenes to evaluate potential synergistic effects. In vivo evaluation (using an animal model of scabies) is also required.

## Data Availability

The data supporting the results of this paper are included in the paper.
